# Ethical and scientific considerations for patient enrollment into concurrent clinical trials

**DOI:** 10.1186/1745-6215-15-470

**Published:** 2014-11-29

**Authors:** Paul S Myles, Elizabeth Williamson, Justin Oakley, Andrew Forbes

**Affiliations:** Department of Anesthesia and Perioperative Medicine, Alfred Hospital, Commercial Road, Melbourne, VIC 3004 Australia; Department of Anesthesia and Perioperative Medicine, Monash University, Melbourne, Australia; National Health and Medical Research Council Practitioner Fellow, Melbourne, Australia; Department of Medical Statistics, London School of Hygiene and Tropical Medicine, London, United Kingdom; School of Public Health and Preventive Medicine, Monash University, Melbourne, Australia; Centre for Human Bioethics, Monash University, Melbourne, Australia

**Keywords:** Autonomy, Co-enrollment, Drug interactions, Ethics, Perioperative medicine, Safety, Statistics, Surgery

## Abstract

Researchers and institutional review boards often consider it inappropriate for patients to be asked to consent to more than one study despite there being no regulatory prohibition on co-enrollment in most countries. There are however ethical, safety, statistical, and practical considerations relevant to co-enrollment, particularly in surgery and perioperative medicine, but co-enrollment can be done if such concerns can be resolved. Preventing eligible patients from co-enrolling in studies which they would authentically value participating in, and whose material risks and benefits they understand, violates their autonomy - and thus contravenes a fundamental principle of research ethics. Statistical issues must be considered but can be addressed. In most cases each trial can be analyzed separately and validly using standard intention to treat principles; selection and other biases can be avoided if enrollment into the second trial is not dependent upon randomized treatment in the first trial; and valid interaction analyses can be performed for each trial by considering the patient’s status in the other trial at the time of randomization in the index trial. Clinical research with a potential to inform and improve clinical practice is valuable and should be supported. The ethical, safety, statistical, and practical aspects of co-enrollment can be managed, providing greater opportunity for research-led improvements in clinical practice.

## Introduction

Large, investigator-initiated randomized clinical trials are able to more fully evaluate the overall effectiveness of new drugs and other treatments in routine practice[1–4]. They are often referred to as ‘practical’ or ‘pragmatic’ trials[2, 3], and are usually publicly funded by national research agencies because they are viewed as ‘public-good’ research[3–5]. There has been a recent upsurge in interest of methods to avoid waste and inefficiency in biomedical research[6]. Obtaining adequate funding for large clinical trials is, however, difficult[7] and they often compete for researcher interest and patient recruitment with local research projects and/or pharmaceutical industry-sponsored trials.

Any patient admitted to hospital for surgery might be eligible for more than one trial, but there is reluctance from many clinicians to co-enroll patients because of ethical, scientific and/or safety concerns. Certainly industry-sponsored trials typically prohibit co-enrollment, and this, therefore, reduces opportunity for investigator-initiated trials. Of greater importance however, is that both researchers and institutional review boards (IRBs) often consider it inappropriate for patients to be asked to consent to more than one study despite there being no regulatory prohibition on co-enrollment in most countries[8, 9].

## Review

Support for co-enrollment in trials has been enunciated in various fields including critical care[8], emergency[9], stroke[10], obstetric[11] and neonatal[11–14] medicine trials. There is also ongoing research concerning co-enrollment in the field of human immunodeficiency virus clinical trials[15]. The Terry Beirn Community Programs for Clinical Research on Acquired Immunodeficiency Syndrome is a clinical trials network funded by the National Institute of Health. This group encourages co-enrollment, and has established procedures to optimize this, including promoting the harmonization of data collection forms across studies and obtaining patients’ consent for multiple clinical trials[16]. Given the complexity of surgical patient comorbidity, potential for drug interactions, and possible overlap of clinical studies done by other disciplines in the perioperative period, co-enrollment into anesthesia, surgery and other perioperative medicine trials need specific consideration.

In this article we consider the ethical, practical, financial and scientific considerations relevant to co-enrollment in our settings and draw conclusions about when and how co-enrollment can be implemented.

### Ethical considerations

National research and drug administration agencies, as well as the World Medical Association Declaration of Helsinki[17], provide ethical guidance to clinical researchers. There are three basic principles that are particularly relevant to the ethics of research involving human subjects: respect for persons, beneficence, and justice[18]. Respect includes acknowledging the rights of an individual to decide whether or not to join a research study they are offered an opportunity to participate in (that is autonomy); beneficence includes both ‘patient-good’ and ‘public-good’ aims of the research; justice refers to fairness and equity of access to healthcare.

Is preventing patients from co-enrolling in two or more clinical trials at the same time ethically justifiable? The autonomy principle underpins the ethical demand to obtain a person’s properly informed consent to participate in a particular study, and to allow them to withdraw from studies at any time[19]. Preventing patients from co-enrolling in studies that they would authentically value participating in, and whose material risks and benefits they understand, would seem to constitute a *restriction* of their autonomy, but such a restriction would only count as *violating* their autonomy when the restriction itself is ethically unjustifiable. Hence, if their participation would compromise the scientific merit or integrity of the study, then excluding them from the study does *not violate* their autonomy. The ethical demand upon investigators to serve the proper goals of research is subject to their also meeting certain crucial side-constraints, such as respecting participant autonomy, and treating participants and others justly. However, where a patient’s autonomous co-enrollment in two or more studies would not compromise the scientific merit or integrity of those studies, and would not involve any injustice to other potential participants (through, for example, causing other relevant patient groups to be under-represented in these studies), then denying them the opportunity to participate in these trials does seem to *violate* their autonomy.

IRBs often restrict or prevent co-enrollment because of concerns about the burdens for patients in participating. To our knowledge, there does not seem to be any empirical research substantiating such an approach. Generally speaking, intervening in the decisions of a competent or decisionally-capable person for their own sake constitutes an exercise of *strong* paternalism, and is regarded by many philosophers as ethically unjustifiable[20]. These actions are distinguished from *weak* paternalistic interventions, aimed at protecting or benefiting people who lack decisional capacity, which are usually seen as ethically justifiable in certain circumstances[21]. Thus, seeking to protect decisionally-capable patients by preventing them from autonomously co-enrolling in two or more studies counts as an exercise of strong paternalism, which is difficult to justify ethically. Some philosophers have argued that strong paternalistic interventions can be ethically justifiable in exceptional cases, where; for example, a person’s autonomous decision at a given time removes their capacity and ability to act autonomously in the future - even John Stuart Mill, the champion of individual liberty, held that it is ethically justifiable to prevent a person from voluntarily selling themselves into slavery[22]. However, there is a heavy burden of proof upon such arguments to demonstrate that such strong paternalistic interventions are necessary in the circumstances to prevent the person altogether relinquishing their future capacity for autonomy. And it is far from clear that co-enrollment in two or more clinical trials, generally speaking, threatens to remove patients’ capacities to act autonomously in the future.

Rejecting general prohibitions on co-enrollment is also consistent with key national guidelines and principles of research ethics, which take beneficence, respect for persons, and justice as central values governing the ethical conduct of research. Such prohibitions are not usually based on concerns about justice to participants, and patients need to have an opportunity to make their own autonomous decisions about whether or not to co-enroll in two more studies which meet the criteria for scientific merit and integrity. Indeed, allowing co-enrollment can enable research to be done more efficiently, which itself can be an important factor in the just allocation of the financial and other resources which are available to investigators to conduct research.

Respect for personal autonomy - the capacity to make one’s own decisions in life according to one’s own values and interests as to what constitutes a meaningful life[19] - is a key ethical requirement. As stated in the Belmont Report in 1979[18], to the degree that research subjects are capable, they should be given the opportunity to choose what shall or shall not happen to them. Investigators cannot be required to withhold information about potential studies from a prospective participant merely because they would entail co-enrollment. It is only after the likely personal and societal benefits, and possible costs and harms, of participating in one or more studies have been clearly communicated to the person that they can make an informed decision about whether to participate in those studies. But it is not only the details of a particular study that need explanation, it is also the existence of such a study - for denial of the opportunity to participate in a study that they wish to participate in and for which they meet the inclusion criteria denies the potential participant autonomy. Potential study participants should be offered the opportunity to decide whether or not a particular research project is too burdensome. Anything less would seem in itself, unethical.

IRB members have a responsibility to protect the interests of study participants, but this does not mean they should decide what is best for participants. A person’s values and motivations deserve consideration and these remain unknown until the person is approached. An additional consideration would be to ask potential study participants to choose their preferred one or more of several current trials[23].

### Perspectives of patients and researchers

Previous studies have demonstrated that potential participants in clinical trials, including parents of children and next-of-kin of critically ill patients, strongly support participation in research[12–14]. Numerous surveys have noted that altruism[12, 24, 25], and perhaps the possibility of receiving a new and potentially superior treatment, are key motivations for participants to enroll in clinical research projects.

Parents of infants receiving intensive care who had either agreed or declined consent to their child’s participation in research were asked about which factors influenced their decision[13]. The most important aspects were the parents’ risk-benefit assessment, their attitude towards research, and the integrity of the consent process. Around one third of the parents preferred the doctors to advise them rather than make an independent decision. In another study, parents of premature infants reported they were mostly comfortable with their child being enrolled in more than one study at any one time[12]. A clear majority (71%) of the parents thought it was very good for their baby to be in a hospital that was carrying out a lot of research. Most (93%) thought that their baby would get the same or better care. Only 22% were worried about the number of studies; and 74% were willing for their baby to participate in 2 or more studies, and 10% would enroll in all 7 proposed studies. Importantly, there was direct support for the public good stance outlined above, in that most parents (94%) believed that their baby’s participation would improve care of future babies.

Cook *et al*.[8] conducted a survey of critical care researchers from the Canadian Critical Care Trials Group and the Australian and New Zealand Intensive Care Society Clinical Trials Group regarding enrollment of critically ill children and adults into one or more studies. They found that only 11% of their respective IRBs had a formal co-enrollment policy and even then, these policies were highly variable.

Interestingly, the lay public seems to be less supportive of enrollment into a randomized trial when compared with actual patients who have a medical condition[26], suggesting that values and preferences of members of an IRB and actual patients may not align. That is, IRB members may be too conservative and thus fail to represent the interests of the potential participants of clinical research. Burden should be primarily determined by the study participant, and this cannot be resolved unless the potential participant is informed of the proposed research.

### Safety considerations

Many investigators believe that if patients are enrolled in two or more clinical studies the effects of each intervention may interfere with one another. Unexpected drug-drug interactions could be potentially unsafe for the co-enrolled study participants. This possibility should always be carefully considered before co-enrollment is undertaken. It will be a concern particularly when two studies are testing drugs that can directly interact with each other (for example, two anticoagulants), and most early-phase drug development studies where the effects and side effects of the investigational drug remain unclear. Of course, such interactions can occur outside the auspices of a randomized trial. Most perioperative and critical care studies enroll patients with several comorbidities treated with a range of co-administered medications. A cursory look at the tabulated baseline characteristics in most perioperative phase III/IV drug trials shows that the many types of medication that participants are taking in addition to the trial drug attests to the potential for interactions. In other words, co-enrollment into several drug trials is little different from what occurs in single-enrollment studies. Adverse drug interactions may occur in any case. There should be clear guidelines for the detection and reporting of adverse events in all drug trials[27].

### Scientific and statistical considerations

The key scientific concern surrounding co-enrollment is that by enrolling patients into two or more trials the scientific validity of the individual trials may be threatened. In particular, it is possible that the effects of the individual interventions could interfere with each other. Such drug-drug interactions could potentially change the effects of each individual intervention, thereby leading to different conclusions from those that would have been drawn if the two trials had been conducted on separate patient populations. A second concern is the potential effect of co-enrollment on the statistical power of the individual trials.

#### Effect of co-enrollment on ability to assess drug-drug interactions

The relevance of the first statistical concern, that of drug-drug interaction, is very dependent on the specific scenario under consideration. Here we are considering the context of phase III/IV pragmatic trials in the field of anesthesia, which are conducted with a heterogeneous population receiving other drug treatments for their underlying condition(s). Clinical trials that allow a broad range of study participants and options for drug treatment(s) and other therapies are highly valued because they provide greater external validity, or generalizability, of the trial results[1, 2, 4]. Phase III/IV trials typically include patients with comorbidities for which drug-disease interactions may occur, as well as a variety of non-drug treatments affecting outcome. The interventions being evaluated tend to be commonly used in practice albeit in a non-randomized fashion, being at the discretion of the anesthesiologist and other treating physicians. These clinical trial participants are therefore exposed to drug and other treatment interactions in a largely uncontrolled and poorly evaluated fashion. In these circumstances, rather than introducing previously absent drug-drug interactions, co-enrollment facilitates evaluation of such interactions, at least to some degree.

A more deliberate approach to the simultaneous evaluation of two or more interventions would be to adopt a factorial design[28]. This is a robust and efficient approach, particularly if there is a low likelihood of interaction between the interventions. Factorial designs are cost-efficient, testing two or more treatments for the price of one[5, 28]. Larntz *et al*.[16] point out that subgroups defined by randomized treatment assignment protect against imbalance or confounding; thus, a factorial design allows unbiased assessment of interaction between the interventions. As with many interaction analyses, these assessments can have low statistical power. However, Lilford *et al*.[5] note that the argument that factorial trials *may* miss interactions between interventions is specious because the alternative is that separate trials *will* miss any such interactions.

A good example of a factorial design in anesthesia is the IMPACT trial testing six anti-emetic interventions[29]. The study was primarily designed to determine the relative benefit of several antiemetic interventions. The 6-way factorial design meant that 8 antiemetic combinations could be evaluated, with 64 (2 × 2 × 2 × 2 × 2 × 2) possible drug-drug combinations. The investigators found that ondansetron, dexamethasone, and droperidol each reduced the risk of postoperative nausea and vomiting by about 25%; propofol reduced the risk by 19% and avoidance of nitrous oxide (replaced with air) 12%. This design allowed the researchers to determine that all the interventions acted independently of one another.

A key question is whether co-enrolled trials can provide some of the same advantages as factorial trials when the enrollment and randomization is *not* concurrent. Clearly, co-enrollment shares the advantage of requiring fewer participants in total. As we will demonstrate, co-enrolled trials also allow the unbiased assessment of interaction between interventions provided that sufficient numbers of participants are co-enrolled. However, extra care is needed in the construction of appropriate interaction analyses compared with a factorial trial.

**Figure 1:**
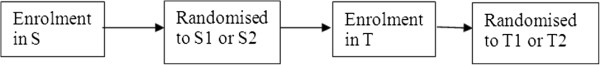
**A schematic representation of the time sequence of enrollment and randomization in the two trials.**

The reason why evaluating drug-drug interactions is not straightforward in co-enrolled trials is that conventional interaction analyses are prone to bias when based on the subset of patients who are enrolled in both trials. This bias arises when the randomized intervention allocated in one trial influences enrollment into a subsequent co-enrolled trial, and enrollment in the subsequent trial depends on health status (as it almost always will). To illustrate this point, we consider a simplified example of two trials, S and T, with enrollment to S always occurring before enrollment to T. Schematically, the time sequence is presented in Figure1.

Suppose patients in Trial S take a range of concomitant medications, some including a Drug X prescribed to patients in generally poor health status. Patients are randomly assigned to one of two treatments, S1 and S2. Due to randomization, the use of Drug X will be balanced between treatment arms S1 and S2. Suppose that the later Trial T has the following determination for entry: either a patient has not taken Drug X, or the patient has received treatment S1 in Trial S. Then, among patients in Trial S who are also enrolled later in Trial T, those who are taking Drug X must necessarily have received treatment S1. This means that among patients enrolled in both trials the patients receiving S1 are less healthy than those receiving S2, thereby ruining the balance in underlying health status in this subgroup that randomization had provided among all Trial S patients. In causal modeling language, this is known as (selection) ‘bias from conditioning on a collider’[30] and has also been discussed in the context of adjustment for post-randomization variables[31]. The consequence of this selection bias is that we cannot validly compare the effect of S1 (compared to S2) between patients who received the active intervention in Trial T, and similarly we cannot compare S1 versus S2 among patients receiving the control intervention in Trial T. We therefore cannot answer the question of whether the difference in effects of the treatments S1 and S2 evaluated in Trial S was modified by the intervention received in Trial T. However, there is a fundamental asymmetry between Trials S and T due to their temporal ordering of enrollment: we can assess whether the difference in effects of the drugs evaluated in Trial T (T1 and T2) was modified by the intervention received in Trial S, since treatment in Trial S is a pre-randomization characteristic at the time of randomization for Trial T.

In co-enrolled trials where participants can be enrolled into either of two trials first, this idea can be extended, showing that the effect-modification of each drug by the other can be assessed, provided that the enrollment and randomization status of the other trial *at the time of randomization into the index trial* is taken into account. This is discussed in detail by Larntz *et al*.[16] in the context of co-enrolled human immunodeficiency virus trials. In brief, we compare the effect of the active drug being evaluated in Trial T between participants who had been randomized to S1 and S2 *at the time of their enrollment to Trial T* (so participants who were not enrolled into Trial S at the time of enrollment into Trial T are not included in this analysis, irrespective of later enrollment into Trial S), and *vice versa*. Therefore, when considering interactions, we need to emphasize the fundamental importance of the status of each patient at the time of randomization in each trial. Patients enrolled and randomized to treatments T1 and T2 in Trial T *after* randomization to Trial S are regarded as *not enrolled* in T when interactions between treatments in Trials S and T are being considered.

Thus, naïve attempts to examine interactions based on *a posteriori* allocated treatments without regard to their timing are prone to selection bias and would need to be justified on substantive grounds why such bias is not plausible. An example where this may be justified is with double-blind trials where there may be little opportunity for allocated treatment in the first trial to influence enrollment into the second trial. However, even in this case side effects from one of the blinded treatments may make participation in the second trial less likely, leading again to selection bias. Furthermore, it may be tempting to also consider trials with apparently simultaneous enrollment of patients as being justified. However, even in such situations a patient must enroll in the trials sequentially, no matter how small the time interval between enrollments. If randomized allocation to the first trial occurs immediately after enrollment and prior to enrollment in the second trial then bias may occur if there is opportunity for that allocation to influence enrollment. The only occasion where there is truly no bias potential is with proper factorial designs, with effectively two trials run together with absolutely concurrent enrollment and randomization.

Our discussion thus far has concerned concepts of interaction and its effects. Also of importance are standards for conducting and reporting analyses where interactions are present, and guidelines for these are available[32, 33].

#### Note on interpretation of results of individual trial analyses in the presence of an interaction

Co-enrollment into two or more trials does not invalidate the original randomization of the individual trials. Separate analysis of each individual trial, ignoring the issue of co-enrollment into the other trial, will retain the balance of patient characteristics expected by standard random assignment within each trial. However, although analysis of an individual Trial S can proceed ignoring the presence of interventions in Trial T, if there are interactions between the interventions of the two trials then the estimated treatment effects in Trial S is a weighted average of the effects at each level of the treatments of Trial T. It therefore estimates the treatment effect in a hypothetical population where patients receive both treatments in the same proportions as in Trial S. This may or may not correspond to any actual patient population of interest depending on how realistic the concurrent treatment usage patterns are in actual practice. As a result the generalizability of the results of the individual trials may potentially be compromised.

#### Effect of co-enrollment on statistical power

We now turn to the second scientific and statistical concern with co-enrollment; the potential effect of co-enrollment on the statistical power of the individual trials. Co-enrollment can change the outcomes from that of the natural course of treatment for patients enrolled in an initial trial. For example, for patients enrolled in Trial S initially, the natural course may be that patients receive later, in non-randomized manner, treatment T1 with probability 20% and treatment T2 with probability 80%. (In practice, these probabilities would depend on patient clinical characteristics). However, if co-enrolled later in Trial T these probabilities shift to 50% due to 1:1 randomization, and therefore if there is differing efficacy of T1 and T2, the outcomes of patients in Trial S in the absence of Trial T will differ to the outcomes in the presence of Trial T. Although intention to treat analysis of Trial S ignoring Trial T status will not be biased for estimating the effect of randomization to treatments S1 versus S2, the power of Trial S will be altered to some degree by the altered prognosis of patients later randomized in Trial T. Note that this altered prognosis of patients would occur even if Trials S and T were run concurrently as in a factorial trial, so this power issue is not at all unique to sequentially co-enrolled trials.

We have examined numerically a number of scenarios of enrollment into an initial Trial S then possible co-enrollment into a second Trial T. The statistical power for treatment comparisons in the initial Trial S depends on a range of factors, including the amount of co-enrollment, the altered prognosis of patients due to the existence of a true effect of either or both treatments T1 and T2, the existence of an interaction between the treatments in Trials S and T1 and T2, the severity of condition of the patient population, the eligibility criteria for Trial T, and the use of treatments T1 and T2 in patients enrolled in S but not enrolled in the second Trial T. General conclusions are not easily summarized due to the wide variety of input parameters. However, for any proposed co-enrollment situation, although investigators cannot change the effects of the two interventions or their interaction, they can potentially control the co-enrollment fraction. Therefore, we investigate the impact of the co-enrollment decision in situations where all design characteristics and effects of treatments in Trial S and T are held fixed. We provide some numerical results here for the following situations which we consider fairly representative of large scale investigator-initiated trials:

-  There is a binary classification of disease severity, and either 60% or 30% of patients in Trial S are classified as severely diseased
-  The probability of enrollment in Trial T does not depend on disease severity of the patient
-  Patients not enrolled in Trial T may receive treatments T1 and T2 of Trial T. We consider 3 cases: (i) treatment T1 is universally used; (ii) treatment T2 is universally used; (iii) patients received treatment T1 with probability according to their disease severity: 30% of severely diseased patients receive treatment T1, 60% of non-severely diseased patients receive T1
-  All patients receive their assigned treatment, with no crossover or dropout
-  The statistical model for event rates is a linear model with interaction, which can be expressed as the probability of a poor outcome for non-severely diseased patients:
  - 7% if receive S2 and T2
  - 5% if receive S1 and T2
  - 6% if receive S2 and T1
  - Add Q% if receive S1 and T1 (amount Q will be varied)
  - And add 3% to each of the above outcome probabilities if the patient is severely diseased
-  We assume the same statistical model for event rates applies regardless of whether patients are co-enrolled in Trial T or not. That is, the event rate depends on which treatment of Trial T is received, regardless of whether or not it is received in Trial T or external to it.

In statistical terms we write the model as:

We consider three values of Q, namely -1%, 0, and +1%, corresponding to a synergistic interaction between the treatments in S and T; no interaction; and an antagonistic interaction, respectively. A synergistic interaction between treatments S1 and T1 means that their combined effect is larger than that predicted from their individual effects. An antagonistic interaction has the combined effect less than predicted from the individual effects. In the above model, the synergism and antagonism are the same size as the effect of treatment T1, and therefore these are quite large effects.

**Figure 2:**
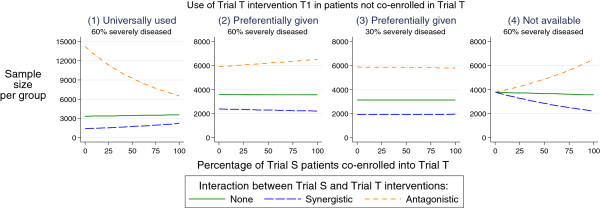
**Sample size per arm to detect a difference between interventions in Trial S with 90% power for 4 scenarios according to co-enrolled fraction in later Trial T.** Scenario (1) corresponds to treatment T1 of Trial T used for all non-co-enrolled patients. Scenarios (2) and (3) correspond to preferential use of T1 in non-co-enrolled patients depending on their severity. Scenario (4) corresponds to no use of T1 in non-co-enrolled patients.

Figure 2 displays the dependence of the required sample size on the co-enrollment fraction for each of the four cases of use of Trial T treatments outside of Trial T. We can provide a general intuitive explanation for why co-enrollment might be expected to produce large or small increases or decreases in required sample size. The main element concerns the amplification or diminishment of the effect of the interaction between the treatments of the two trials according to the frequency of use of treatment T1 in the *non*-co-enrolled patients of Trial S, that is, the frequency of use of treatment T1 *outside* of Trial T.

First, synergistic interactions tend to increase statistical power (and hence have reduced sample size requirements), whereas antagonistic interactions decrease power[32]. Therefore, the effects of co-enrollment on power will depend both on the direction of the interaction, and also on whether co-enrollment increases or decreases the ‘exposure’ of patients in Trial S to the receipt of treatment T1 of Trial T, and hence to the interaction. The greater the proportion of patients in Trial S exposed to treatment T1, the greater the effects of the interaction.

This can be summarized succinctly: if co-enrollment increases the proportion of patients in the first Trial S receiving treatment T1, then an antagonistic interaction will increase required sample size for the first trial, but a synergistic interaction will decrease sample size. Scenario (4) of Figure 2 displays this case - with zero co-enrollment and no exposure to treatment T1 outside of Trial T, then no patients in Trial S receive treatment T1 and interaction has a moot null effect. If there is 100% co-enrollment then all patients in Trial S receive treatment T1 and the interaction has maximal effect on Trial S patients in the directions anticipated by synergism and antagonism.

Analogously, if co-enrollment decreases the proportion of patients in the first Trial S receiving T1 then an antagonistic interaction decreases the required sample size and a synergistic interaction increases sample size. This is displayed in Scenario (1) of Figure 2 where zero co-enrollment means all patients in Trial S are treatment with treatment T1 and hence maximal interaction effects are observed. With 100% co-enrollment, then only 50% of patients are exposed to T1 (because of randomization in Trial T) and hence the effect of the interaction is reduced.

For intermediate scenarios, such as Scenarios (2) and (3) of Figure 2, the effects of co-enrollment on sample size depends on the exposure of non-co-enrolled patients to T1 outside of Trial T relative to that of 50% exposure for co-enrolled patients (because of randomization in Trial T). If non-co-enrolled exposure to T1 is greater than 50% then the greater the co-enrollment the less the overall exposure of patients of Trial S is to treatment T1 and hence the less the impact of the interaction. Conversely, if non-co-enrolled exposure to T1 is less than 50%, then the greater the co-enrollment the greater the impact of the interaction.

In the Appendix we show that for Scenario (2) of Figure 2 the non-co-enrolled exposure to T1 is 42%, and hence the greater the co-enrollment the greater the impact of the synergistic and antagonistic interactions. However, these effects are rather mild, with the synergistic interaction yielding at most a 9% increase in sample size required.

For Scenario (3) of Figure 2, the Appendix shows that the non-co-enrolled exposure to T1 is 51%, and therefore non-co-enrolled patients are receiving T1 at the same proportion as co-enrolled patients and co-enrollment has negligible impact on sample size.

More generally, we have found empirically that for studies with primary endpoints in the range of 5% to 10% that co-enrollment has a small effect on study power, mostly resulting in less than a 20% difference between smallest and largest sample sizes. However, we caution that this need not always be the case - in the Appendix we describe situations with rather extreme antagonistic interactions where co-enrollment may have a substantial impact on sample size.

Therefore, for the simplified setting considered above of an initial Trial S followed by potential co-enrollment in Trial T, for co-enrollment to have a large detrimental effect on the sample size of the initial Trial (S), there needs to be a large antagonistic interaction, substantial co-enrollment, and patients not co-enrolled receive treatment T1 at a lower frequency than 50%. Whether this is likely to occur will of course depend on the particular clinical circumstances of the trials and treatments under consideration.

We now summarize all the statistical issues discussed above:

If it is reasonable to assume that:

(i) The randomized treatment in the first trial has not influenced enrollment into the second trial, and
(ii) That neither treatment influences the natural course of disease of the other condition being studied, and
(iii) That there is unlikely to be a drug-drug interaction,

then the scientific validity of the individual trials should not be compromised or complicated by co-enrollment. Violation of any of these conditions means that careful consideration of the issues explained above is required.

### Practical and cost-effectiveness considerations

Clinical research with a potential to inform and improve clinical practice is valuable and should be supported[2, 3, 10, 34]. It has often been noted that there is a lost opportunity in most areas of clinical practice, because a majority of patients are not participating in clinical trials.

A select few departments and/or institutions have established clinical research infrastructure, and often lead multinational, multicenter trials that provide funding, typically on a per patient basis, to numerous other ‘secondary’ sites. These secondary sites are dependent on patient recruitments to fund their local research staff, but this research income does not necessarily cover costs. Opportunities for local sites to co-enroll patients into a number of projects could generate higher income for them and so bolster their involvement in research. More studies can be done in a timely fashion and more information to guide clinical practice can be generated[3, 6, 35, 36].

## Conclusion

Overall, the ethical, scientific and safety aspects of co-enrollment can generally be managed, providing greater opportunity for research-led improvements in clinical practice. Researchers should consider the various research projects for which patients may be eligible. The main issues to consider are whether study-related procedures might increase study burden on the participants, the impact that co-enrollment might have on study power and on the potential for selection bias of interaction analyses.

## Appendix

### Details of the computation of sample size according to co-enrollment fraction

Let N denote the total number of patients in Trial S. Of these N patients, a proportion will be co-enrolled in Trial T, and the remainder will receive one of the treatments of Trial T (without being co-enrolled in Trial T) with probability according their severity.

Let *P*_*ijcs*_ be the outcome probability for a patient who receives treatment *i* in Trial S (i = 1 if S1, i = 2 if S2), treatment j of Trial T (j = 1 if T1, j = 2 if T2), has co-enrollment status c (c = 1 if co-enrolled in both trials, c = 2 if enrolled in Trial S only), and is of underlying severity s (s = 1 for severe, s = 2 for non-severe).

Because the outcome probability only depends only on treatments S and T received and patient severity, regardless of whether the patients are actually co-enrolled in Trial T or not, *P*_*ij*1*s*_ = *P*_*ij*2*s*_.

Let *n*_*ijcs*_ be the number of patients receiving treatment *i* in Trial S, treatment j of Trial T, are of co-enrollment status c, and are of severity s.

Note that due to randomization, co-enrolled patients are allocated to each combination of treatments S (1,2) and T (1,2) with probability 1/4. Also note that for patients not co-enrolled, since the probability of their receipt of treatments T1 or T2 depends solely on their severity and not on treatment received in Trial S, 1/2 of patients receiving treatment T1 will have received treatment S1 and 1/2 will have received S2, and similarly for patients receiving T2.

Therefore, for co-enrolled patients:

And for non-co-enrolled patients:

Then the marginal event rate for each treatment i of Trial S is

### Sample size formula

Once marginal event rates P_1_ and P_2_ for Trial S have been determined, the sample size per arm of Trial S is then[37]:

where and and *Z*_*β*_ are quantiles of the standard normal distribution for type I error rate *α* and power 1 - *β*.

### Calculation of the probability of non-co-enrolled patients receiving treatment T1 of Trial T

Using the specified probabilities for non-co-enrolled patients receiving treatment T1 of Trial T in the example in the main text, where Pr(T1 = 1 | Severe) = 0.30, and Pr(T1 = 1 | Non-severe) = 0.60, there are two cases:

(i) 60% of patients are severely diseased
(ii) 30% of patients are severely diseased

For these cases:

(i) Pr(Severe) = 0.60:
(ii) Pr(Severe) = 0.30:

### Numerical examples of effect of co-enrollment

Here we provide additional numerical examples of the effect of co-enrollment on sample size under varying conditions of size of antagonistic interaction and proportion of patients who are severely diseased:

- Table  1 above reports the results of calculations with the same model as in the main text but with an antagonistic interaction of 2%, representing a large qualitative interaction where the effect of S1 in the presence of T1 is 0% (that is neutral), and in the absence of T1 is –2%, that is benefit). We note the sample size differential between no co-enrollment and full co-enrollment is approximately 35% (= 15,097/11,219).
- Table  2 extends this further to the case where the antagonistic interaction is +3%: the effect of S1 in the presence of T1 is +1% (that is harm), and in the absence of T1 is –2% (benefit). The sample size differential between no co-enrollment and full co-enrollment is now 120%, owing to convergence of the event rates and full co-enrollment reducing the difference in event rates by 33%.
- Table  3 also considers an antagonistic interaction of 3% but now considers a population where 30% of patients are severely diseased. Here we see a different outcome - there is little change in required sample size as co-enrollment increases, and in fact there is a reduction in sample size of 12%. This difference compared to Table  2 is explained in the main text - it is simply because the exposure fraction of non-co-enrolled patients to treatment T1 is approximately 50% in this case, compared to 42% in Table  2.

**Table 1:** **Co-enrollment fractions and resultant sample size with 2% antagonistic interaction**

Co-enrollment fraction	Event rate for arm S1	Event rate for arm S2	Difference	Number of patients per arm for 90% power
0%	7.22	8.38	-1.16	11,219
30%	7.24	8.36	-1.11	12,209
50%	7.26	8.34	-1.08	12,943
70%	7.28	8.32	-1.05	13,746
100%	7.30	8.30	-1.00	15,097

**Table 2:** **Co-enrollment fractions and resultant sample size with 3% antagonistic interaction**

Co-enrollment fraction	Event rate for arm S1	Event rate for arm S2	Difference	Number of patients per arm for 90% power
0%	7.64	8.38	-0.74	28,249
30%	7.69	8.36	-0.67	34,715
50%	7.72	8.34	-0.62	40,335
70%	7.75	8.32	-0.57	47,432
100%	7.80	8.30	-0.50	62,161

**Table 3:** **Co-enrollment fractions and resultant sample size with 2% antagonistic interaction and an at-risk study population**

Co-enrollment fraction	Event rate for arm S1	Event rate for arm S2	Difference	Number of patients per arm for 90% power
0%	6.92	7.39	-0.47	63,137
30%	6.91	7.39	-0.48	60,775
50%	6.91	7.40	-0.48	59,272
70%	6.91	7.40	-0.49	57,825
100%	6.90	7.40	-0.50	55,751

## Abbreviations

IRB: institutional review board
Pr: probability.
